# Integrated transcriptome-microbiome analysis reveals a host-microbe interplay associated with insecticide resistance in *Aedes albopictus*

**DOI:** 10.3389/fmicb.2026.1788609

**Published:** 2026-04-22

**Authors:** Lifang Liu, Guorui Liang, Heting Gao, Siyu Xing, Kai Wang, Xinyu Zhou, Xinan Huang, Chunxiao Li

**Affiliations:** 1 Artemisinin Research Center, Guangzhou University of Chinese Medicine, Guangzhou, China; 2 State Key Laboratory of Pathogen and Biosecurity, Beijing, China

**Keywords:** *Aedes albopictus*, beta-cypermethrin, gut microbiome, insecticide resistance, transcriptome

## Abstract

**Introduction:**

*Aedes albopictus* is the primary vector of major arboviral diseases such as dengue fever, chikungunya fever, and Zika virus disease, and its control is highly dependent on chemical insecticides. However, the long-term use of pyrethroid insecticides has led to the development of insecticide resistance in *Ae. albopictus*, which severely undermines the efficacy of vector control programs.

**Methods:**

*Ae. albopictus* populations were collected from five sites in Guangdong and Hainan provinces, China. Beta-cypermethrin resistance levels were determined via bioassays, with resistance ratios at the median lethal concentration (RR_50_) calculated. Target-site resistance was evaluated via *kdr* mutation detection in the voltage-gated sodium channel (VGSC) gene. Transcriptome sequencing identified differentially expressed genes (DEGs), and 16S rRNA sequencing characterized gut microbiome alterations. Correlation analysis and *Cedecea neteri* dietary supplementation assays verified the role of gut microbiota in resistance.

**Results:**

The results showed that all four populations (except the CP population) exhibited varying degrees of resistance to beta-cypermethrin, with resistance ratios at the median lethal concentration (RR_50_) ranging from 2.84 to 29.18. Detection of *kdr* mutations revealed three mutations (F1534C, F1534L, F1534S) at codon 1534 of the voltage-gated sodium channel (VGSC) gene in all field populations, with mutation frequencies ranging from 49.4% to 100.0%, and a low-frequency V1016G mutation at codon 1016. Transcriptome analysis identified a total of 2,566 commonly upregulated genes and 994 commonly downregulated genes across the resistant populations. Gut microbiome analysis revealed a significant alteration in the intestinal microbial community structure of resistant populations; specifically, the relative abundance of the genus *Cedecea* differed significantly between resistant and susceptible populations and correlated strongly with the expression of most differentially expressed genes. Furthermore, dietary supplementation with Cedecea neteri significantly increased the survival rate of *Ae. albopictus* exposed to β-cypermethrin (73.86% vs 40.00%; *P* < 0.0001).

**Discussion:**

From the perspectives of target-site mutations, gene expression regulation, and gut microbe interactions, this study providing a foundation for further studies on resistance mechanisms in *Ae. albopictus*, thereby providing a theoretical foundation for further dissection of resistance mechanisms and optimization of vector control strategies.

## 1 Introduction

*Ae. albopictus* is the primary vector for multiple insect-borne infectious diseases, such as dengue, Zika, and Chikungunya, and is also a global invasive species (Benelli et al., 2020; Zhang et al., 2023). Currently, there are no specific medications or vaccines for mosquito-borne diseases like dengue fever (Wu et al., 2023; Huang et al., 2023). Vector control relying on chemical insecticides remains the primary prevention and control method, with pyrethroid insecticides being particularly widely used (Wilson et al., 2020; van den Berg et al., 2021, 2012). However, long-term large-scale use has led to resistance in *Ae. albopictus*, limiting the effectiveness of prevention and control measures (Dusfour et al., 2019; Gouagna et al., 2020; Maciel-de-Freitas et al., 2014). In-depth analysis of its resistance mechanism has become an urgent scientific research and prevention and control need.

Traditional studies on insecticide resistance have mostly focused on a single perspective of specific targets or metabolic pathways, making it difficult to comprehensively elucidate the complex resistance phenotypes in field populations (Zalucki and Furlong, 2017; Bass et al., 2015). Target-site mutations in the voltage-gated sodium channel (VGSC) gene, known as *kdr* mutations, are a classic mechanism of pyrethroid resistance, with substitutions at codons 1,016 and 1,534 being the most common and impactful (Xu et al., 2016; Kasai et al., 2019). In recent years, the development of systems biology approaches has provided a new avenue for multi-dimensional dissection of resistance mechanisms. Transcriptomics can globally reveal resistance-related gene expression regulatory networks, including multiple pathways such as detoxification metabolism and stress response (Muthu Lakshmi Bavithra et al., 2023; Xu et al., 2016; Aguirre-Obando et al., 2022). As the “second genome,” the gut microbiome has been proven to affect the metabolic capacity and immune status of the host, and even participate in the direct degradation of insecticides (Kikuchi et al., 2012; Pavlidi et al., 2018; Zhu et al., 2023; Wang et al., 2022; Omoke et al., 2021). Therefore, integrating transcriptome and gut microbiome analyses to systematically illustrate the mechanisms underlying resistance development from the two aspects of host gene regulation and symbiotic microbiota interaction holds great scientific significance and application prospects.

Integrating *kdr* mutation detection, transcriptome, and gut microbiome analyses to systematically explore resistance-associated mechanisms from the three aspects of target-site mutations, host gene regulation, and symbiotic microbiota interaction holds great scientific significance and application prospects. In this study, we collected field populations of *Ae. albopictus* from five geographic locations in Guangdong and Hainan provinces, China, with varying levels of resistance to β-cypermethrin. We first quantified the resistance levels of each population using the CDC bottle bioassay and detected *kdr* mutations in the VGSC gene. We then used transcriptomic sequencing and full-length 16S rRNA amplicon sequencing to characterize changes in host gene expression and gut microbial community structure. By correlating these multi-omics datasets, we identified key genes, mutations, and microbial taxa linked to the resistance phenotype and verified the role of a candidate microbe (*Cedecea neteri*) in resistance through functional experiments. This work aims to provide a comprehensive molecular foundation for understanding the development of β-cypermethrin resistance in *Ae. albopictus* and guide the optimization of future vector control strategies.

## 2 Materials and methods

### 2.1 Field collection of mosquitoes and rearing of the F1 generation

From late June to early July 2023, adult *Ae. albopictus* mosquitoes were collected using the double-layer bed net method recommended by the World Health Organization at three sampling sites in Guangzhou (Haizhu Lake Area, Yuexiu Park, Liwan Lake Park) and two sampling sites in Haikou, Hainan Province (Wanlv Garden, Haikou Shishan Volcanic Cluster Global Geopark). After field collection, all adult mosquitoes were immediately transported to the laboratory, anesthetized with carbon dioxide, and kept on ice. Species-level morphological identification was performed under a stereomicroscope according to the taxonomic keys and morphological descriptions in *Fauna Sinica: Insecta Vol. 8 Diptera: Culicidae I* (Lu, 1997). Specimens morphologically confirmed as *Ae. albopictus* were pooled and reared under standard laboratory conditions (temperature: 27 ± 2°C; relative humidity: 75 ± 5%; photoperiod: 16 h light: 8 h dark). Adults were provided with blood meals to induce oviposition. Egg batches were collected and hatched, and larvae were reared to adulthood to obtain F1 generation adults for all subsequent experiments. The susceptible strain (SS) used in this study has been maintained in the laboratory for at least 10 years without exposure to any insecticides, ensuring its susceptibility to β-cypermethrin.

### 2.2 Resistance biological assay of adult mosquitoes

This study adopted the CDC bottle bioassay in accordance with the method recommended by the World Health Organization (WHO) and the official technical guideline of the Centers for Disease Control and Prevention (CDC), *Guideline for Evaluating Insecticide Resistance in Vectors Using the CDC Bottle Bioassay* (2009). Healthy female mosquitoes aged 3–5 days post-emergence from the F1 generation of each population were selected to determine their susceptibility to β-cypermethrin (Aladdin, Cat. No.: C109960). Based on the results of preliminary pre-experiments, six gradient concentrations of β-cypermethrin were set (see Table 1). The exposure time of mosquitoes to the insecticide was 1 h, and the number of dead mosquitoes in each bottle was recorded immediately after the 1-h exposure. Each concentration was set with 3 biological replicates, with 30 adult mosquitoes per replicate. Experimental results were analyzed using GraphPad Prism 8.0.1 software. After log-transformation of the insecticide concentrations, the median lethal concentration (LC_50_) and its 95% confidence interval (95% CI) were calculated by non-linear fitting of the dose-response curve. The resistance ratio (RR) was calculated as RR = LC_50_ of the field population / LC_50_ of the susceptible strain (SS). In accordance with the method described by Wheeler et al. (2006), as well as the relevant specifications of the Ministry of Health of the People's Republic of China and Standardization Administration of China (2011), the criteria for classifying resistant populations were defined as follows: a mosquito population was considered to have significant resistance to β-cypermethrin when the RR50 value was ≥ 5 and its 95% CI did not contain the value 1.

**Table 1:** Testing concentration of β-cypermethrin in CDC bottles of *Ae. albopictus* in various regions.

Area	Concentration 1 (mg/mL)	Concentration 2 (mg/mL)	Concentration 3 (mg/mL)	Concentration 4 (mg/mL)	Concentration 5 (mg/mL)	Concentration 6 (mg/mL)
SS	0.0015	0.003125	0.00625	0.0125	0.0250	0.0500
HZ	0.0250	0.0500	0.1000	0.1500	0.3000	0.9000
YX	0.0250	0.0500	0.1000	0.1500	0.3000	0.9000
LW	0.0125	0.0250	0.0500	0.1000	0.1500	0.3000
CP	0.0125	0.0250	0.0500	0.1000	0.1500	0.3000
WP	0.00625	0.0125	0.0250	0.0500	0.1000	0.1500

### 2.3 Detection of *kdr* resistance alleles

Mosquitoes were randomly sampled from the F1 generation of six *Ae. albopictus* populations. From each population, 3–5 healthy females with no insecticide exposure during rearing were selected,and about 80 individuals per population were used for *kdr* allele detection. Genomic DNA was extracted using a commercial DNA extraction kit (Takara, Japan) following the manufacturer's instructions. DNA quality and concentration were assessed using a NanoDrop Lite Plus spectrophotometer (Thermo Fisher Scientific, USA). Extracted DNA was stored at −20°C or immediately used for PCR amplification.

Specific fragments of the VGSC gene covering codons 1,016 and 1,534 were amplified using primers designed based on previously published sequences (Kasai et al., 2011) (synthesized by Sangon Biotech, Shanghai, China) (Table 2). PCR amplification targeted the transmembrane domains II and III of VGSC, with expected product sizes of 480 bp and 740 bp, respectively. The PCR reaction mixture (50 μL) consisted of 3 μL DNA template, 1 μL of each primer, 20 μL nuclease-free water, and 25 μL Premix Taq™ (TaKaRa, Japan). PCR cycling conditions were as follows: initial denaturation at 95 °C for 5 min; 35 cycles of 94 °C for 10 s, 50 °C for 10 s, and 72 °C for 1 min; followed by a final extension at 72 °C for 5 min. PCR products were verified by 1% agarose gel electrophoresis and subsequently sent for Sanger sequencing (Tianyi Huiyuan Biotech, China).

**Table 2:** Primers for amplification of VGSC gene fragment of *Ae. Albopictus*.

Amplicon	Primer name	Mutation site	Primer sequence (5^′^ → 3^′^)	Fragment length/bp
Domain II	aegSCF20 aegSCR21	V1016	gacaatgtggatcgcttccc gcaatctggcttgttaacttg	480
Domain III	aegSCF7 aegSCR7	F1534	gagaactcgccgatgaactt gacgacgaaatcgaacaggt	740

Sequenced fragments were aligned and compared with reference sequences of *Ae. albopictus* VGSC transmembrane domains II and III (GenBank accession numbers KC152045.1 and KC152046.1) using SnapGene software (GSL Biotech, USA). Nucleotide variations at codons 1016 and 1534 were identified to determine allele types and genotypes. *KDR* allele frequencies and genotype distributions were calculated for each population to evaluate the prevalence of resistance-associated mutations.

### 2.4 RNA extraction, library construction and transcriptome sequencing

Eighty female *Ae. albopictus* aged 3–5 days post-emergence were randomly selected from the F1 generation of six field populations and a laboratory susceptible strain, respectively. No insecticide treatment was performed in the early stage, and samples were taken directly. Four biological replicates were set up for each geographical strain, with 20 mosquitoes per replicate. The samples were quickly frozen in liquid nitrogen and then stored at −80°C. Total RNA was extracted using the TRIzol method, and after quality verification, mRNA was enriched and fragmented with Oligo(dT) magnetic beads to synthesize double-stranded cDNA. Libraries were constructed through end repair, dA-tailing, adapter ligation and PCR amplification, followed by 150 bp paired-end sequencing on the Illumina NovaSeq 6,000 platform.

### 2.5 Bioinformatics analysis of transcriptome

After quality control of the raw data, the sequences were mapped to the *Ae. albopictus* reference genome (version GCF_006496715.2, a_albo_primary_1; accession number: GCF_006496715.2), which is publicly available in the NCBI database. HISAT2 (v2.0.5) software was used for sequence mapping with default parameters; subsequently, featureCounts (v1.5.0-p3) software was used with the same default parameters to perform gene-level quantification of uniquely mapped reads, obtaining raw expression counts.

Prior to differential expression analysis, low-expression genes were filtered out with the following criteria: only genes with raw reads counts ≥ 10 in at least 50% of the samples were retained. This step was implemented using the filterByExpr function in DESeq2 software to eliminate weak expression noise. Differential expression analysis was exclusively performed using DESeq2 (v1.20.0) software, and the median-of-ratios method built into DESeq2 was adopted for normalization. The filtering criteria for differentially expressed genes (DEGs) were set as FDR < 0.05 and |log_2_FC| > 1. Principal Component Analysis (PCA) was used to assess batch effects on the normalized expression data. Gene Ontology (GO) functional annotation and Kyoto Encyclopedia of Genes and Genomes (KEGG) pathway enrichment analysis were performed on the differentially expressed genes (DEGs).

To evaluate the transcriptional contribution of target-site resistance, the expression level of the *kdr* gene (VGSC, gene ID: LOC109421922) was extracted from the normalized expression matrix obtained from DESeq2. The counts of *kdr* transcripts were summarized for each population, and mean expression values were calculated for comparison with the susceptible strain (SS). Log_2_ fold changes and statistical significance (*p*-value and adjusted *p*-value) for *kdr* expression were obtained from the DESeq2 differential expression results. This allowed assessment of whether transcriptional variation of *kdr* could contribute to β-cypermethrin resistance alongside sequence-based target-site mutations.

### 2.6 Library preparation and 16S rRNA sequencing of mosquito gut microbiome

Forty female *Ae. albopictus* (3–5 days after eclosion) were selected from the F1 generation of each population and divided into 4 replicate groups with 10 mosquitoes in each group. The sampled mosquitoes had not been treated with any insecticides in the early stage. To eliminate surface microbial contamination, the mosquitoes were immersed in 75% ethanol for 3 min, followed by rinsing 3 times with PBS buffer. Subsequently, the mosquitoes were placed on sterile glass slides and dissected with sterilized dissecting needles under a stereomicroscope. All dissection operations were performed in a sterile ultra-clean workbench, and the experimental dissecting tools (dissecting needles, tweezers, etc.) were sterilized by high-pressure steam. A blank negative control (only sterile PBS buffer was added without midgut tissue) was set up during the experiment, and this control was subjected to DNA extraction, sequencing and analysis synchronously with the samples to exclude potential environmental and operational contamination during the experiment. The midgut tissues were aseptically dissected and pooled into a sterile 1.5 mL centrifuge tube. Then, the total genomic DNA of the mosquito midgut was extracted by the sodium dodecyl sulfate (SDS) method. After extraction, the purity and integrity of the extracted DNA were verified by 1% agarose gel electrophoresis. A clear, intact DNA band without obvious degradation indicated good DNA integrity; meanwhile, a spectrophotometer was used to quantify the DNA concentration to ensure it met the requirements of subsequent experiments. Qualified DNA samples were diluted to a final concentration of 1 ng/μL with sterile ultrapure water and stored at an appropriate temperature for subsequent PCR amplification and library construction. The V3-V4 region of the 16S rRNA gene was amplified by PCR (F: 5′- AGRGTTYGATYMTGGCTCAG-3′; R: 5′-RGYTACCTTGTTACGACTT-3′). The PCR amplification system consisted of Phusion^®^ High-Fidelity PCR Master Mix (New England Biolabs) containing GC buffer and high-efficiency high-fidelity DNA polymerase to ensure amplification efficiency and accuracy; the PCR program was set according to the high-fidelity amplification conditions recommended by the kit manufacturer. After PCR amplification, the SMRT Bell library was constructed, which mainly included three key steps: adapter ligation and initial purification, fragment size selection and secondary purification, and library quality control. The specific details are as follows: 1. Adapter Ligation and Initial Purification: DNA ligase was used to ligate sequencing adapters to both ends of the purified PCR products (targeting the V3–V4 region of the 16S rRNA gene). Subsequently, AMPure PB magnetic beads were used to purify the ligation products, which could effectively remove unligated adapters, short fragments and other impurities, ensuring the purity of the ligation products. 2. Fragment Size Selection and Secondary Purification: The purified ligation products were redissolved in an appropriate buffer, and the BluePippin system was used to select target products with a specific insert size (matching the amplified fragment of the V3–V4 region). After size selection, AMPure PB magnetic beads were used again for secondary purification to obtain high-quality library fragments with uniform size distribution, which is crucial for ensuring the quality of PacBio SMRT sequencing. 3.Library Quality Control and Sequencing: The concentration of the constructed SMRT Bell library was determined by a Qubit fluorometer, and the insert size was verified by an Agilent 2,100 Bioanalyzer. Only libraries that passed strict quality control (both concentration and fragment size met the sequencing requirements) could be loaded onto the PacBio platform for subsequent sequencing.

### 2.7 Analysis of mosquito gut microbiota community diversity

For microbiome data analysis, consensus sequences were obtained, samples were distinguished by barcode, primers were removed, and sequences were clustered into operational taxonomic units (OTUs) at a 97% similarity level for species annotation. Alpha diversity indices including Chao1 and Shannon were calculated, and beta diversity analyses were performed using principal coordinate analysis (PCoA) and non-metric multidimensional scaling (NMDS).

### 2.8 Correlation analysis between mosquito gene expression and gut microbiota

To further explore the effects of potential interactions between gene expression and gut microbial community of *Ae. albopictus* on its resistant phenotype, multi-omics correlation analysis was performed on gene expression and gut microbiota. Models including Spearman correlation were used to calculate the correlation between the expression levels of the screened differentially expressed genes (DEGs) and the abundance of each differential microbial genus. Mantel test analysis was conducted for drug metabolism-related genes and dominant bacterial genera.

### 2.9 Verification of the correlation between mosquito gut microbiota and pyrethroid resistance in *Ae. albopictus*

Based on 16S rRNA sequencing results, microbial genera showing significant differences between pyrethroid-resistant and susceptible strains were screened. Correlation analysis with the expression levels of differentially expressed genes identified a single bacterial strain, Cedecea neteri, as a candidate for functional testing. The bacterial strain (Cedecea neteri, ATCC 33855) was obtained from the American Type Culture Collection (ATCC). The species identity of the strain was verified and confirmed by ATCC, with the product specification sheet provided by ATCC used as the identification basis. No additional molecular or biochemical identification was performed. The freeze-dried strain was reactivated according to the ATCC product instructions. Bacterial concentration was determined using the standard plate spread method: the log-phase culture was serially diluted (10^−6^ to 10^−9^), and 90 μL of each dilution was spread on trypticase soy agar plates in triplicate. Plates containing 30–300 colonies were selected to calculate CFU using the formula: CFU/mL = colony count/0.09 mL × dilution factor. The culture was then diluted to the experimental feeding concentration (OD_600_ = 0.6–0.8) and re-verified by plate counting to ensure accuracy.

Female mosquitoes aged 3–5 days post-emergence were starved of sucrose for 8 h prior to the experiment. For each experimental group, 30 mosquitoes were used per replicate, with three biological replicates performed. Mosquitoes were fed with the prepared bacterial suspension in 5% sterile sucrose solution for 24 h, while the control group received an equal volume of sterile sucrose solution alone. Following bacterial supplementation, mosquitoes were exposed to 0.1 mg/mL β-cypermethrin using the CDC bottle bioassay. Two sets of data were recorded: (1) Knockdown rate: The number of knocked-down mosquitoes was counted every 10 min during the 1-h exposure period. (2) Survival/mortality: After the 1-h exposure, mosquitoes were transferred to recovery cups, and the survival rate of each group was recorded at 24 h.

### 2.10 Data analysis

Statistical analyses were performed using GraphPad Prism 8.0.1.Kaplan–Meier survival curves were constructed for mosquitoes within 1 h of exposure to beta-cypermethrin, and comparisons between groups were conducted using the Log-rank test. After 24 h of recovery following exposure, survival rates in the treatment and control groups were analyzed using Fisher's exact test. *P* < 0.05 was considered statistically significant.

## 3 Results

### 3.1 Resistance levels of field populations of *Ae. albopictus*

The susceptibility of five field-collected *Ae. albopictus* populations to β-cypermethrin is shown in Table 3. The LC_50_ values ranged from 5.40 mg/L (CP: Haikou Shishan Volcanic Cluster Global Geopark) to 55.44 mg/L [HZ: Haizhu Lake Area (Guangzhou)]. Corresponding RR_50_ values ranged from 2.84 to 29.18, and their 95% confidence intervals (95% CI) were calculated to assess statistical significance (Table 3). The HZ population exhibited the highest resistance (RR_50_ = 29.18), whereas the CP population was relatively susceptible (RR_50_ = 2.84). The relevant guidelines of the Ministry of Health of the People's Republic of China, and Standardization Administration of the People's Republic of China, 2011, populations are considered to have significant resistance to β-cypermethrin if the 95% CIs of their RR50 values do not contain the value 1 and the RR50 values are ≥ 5. Based on this cri terion, the HZ, LW [Liwan Lake Park (Guangzhou)], YX [Yuexiu Park (Guangzhou)] and WP [Wanlv Garden (Haikou)] populations were significantly resistant, while the CP population was considered relatively susceptible.

**Table 3:** Resistance level of five *Ae. albopictus* strains to β-cypermethrin.

Parameters	SS	HZ	YX	LW	CP	WP
LC50 (mg/L)	1.90	55.44	33.00	15.00	5.40	12.47
95% CI of LC50	1.60–2.20	48.25–62.74	28.26–38.80	8.63–22.81	3.36–7.50	10.88–14.24
^a^RR50	1.00	29.18	17.17	7.89	2.84	6.56
95% CI of RR50	1.00	21.93–38.87	12.84–22.90	3.92–14.26	1.53–4.69	4.94–8.70
Phenotypic indication	susceptible	resistance	resistance	resistance	susceptible	resistance

95% CI refers to 95% confidence interval; ^a^RR50: resistance ratio, LC50 test population/LC50 laboratory-susceptible strain.

### 3.2 Detection of *kdr* mutations in *Ae. Albopictus*

Sequencing of PCR products revealed three mutations at codon 1,534 of the *kdr* gene, including F1534C, F1534L, and F1534S (Table 4). Mutations at codon 1,534 were detected in all five field populations, with mutation frequencies ranging from 49.4% to 100.0%. The HZ population exhibited the highest mutation frequency (100.0%), whereas the CP population showed the lowest frequency (49.4%). Among the three mutant forms, F1534S and F1534C were predominant, while F1534L was rare. No mutation at codon 1,534 was detected in the susceptible strain SS.

**Table 4:** Alleles and genotypes at codon 1,534 of *kdr* mutation gene in *Ae. albopictus* various regions.

Area	*N*	Genotypes							Mutation frequency(%)	Allelic frequency(%)		
		**F/F**	**F/C**	**C/C**	**F/L**	**L/L**	**F/S**	**S/S**		**C**	**L**	**S**
HZ	92	0	23	51	0	0	13	5	100	67.9	0	12.5
YX	95	16	24	15	4	0	21	15	83.2	28.4	2.1	26.8
LW	94	15	26	13	4	0	16	20	84.0	27.7	2.1	29.8
CP	77	39	9	0	0	0	26	3	49.4	5.8	0	22.7
WP	88	8	13	12	6	1	44	4	90.9	21.0	4.5	29.5
SS	94	94	0	0	0	0	0	0	0	0	0	0

At codon 1,016, only the V1016G mutation was detected (Table 5). The mutation frequency ranged from 0% to 20.7% among field populations, with the highest frequency observed in HZ. No mutation was detected in WP or SS. Overall, mutations at codon 1,534 were much more prevalent than those at codon 1,016, suggesting that codon 1,534 substitutions may play a more important role in β-cypermethrin resistance in the tested populations.

**Table 5:** Alleles and genotypes at codon 1,016 of *kdr* mutation gene in *Ae. albopictus* various regions.

Area	*N*	Genotypes			Mutation frequency(%)	Frequency of 1016G (%)
		V/V	V/G	G/G		
HZ	92	73	17	2	20.2	11.4
YX	95	82	12	1	13.7	7.4
LW	94	82	11	1	12.8	6.9
CP	77	76	1	0	1.3	0.6
WP	88	88	0	0	0	0
SS	94	94	0	0	0	0

### 3.3 Quality assessment of transcriptome sequencing data and analysis of sample relationships

**Figure 1:**
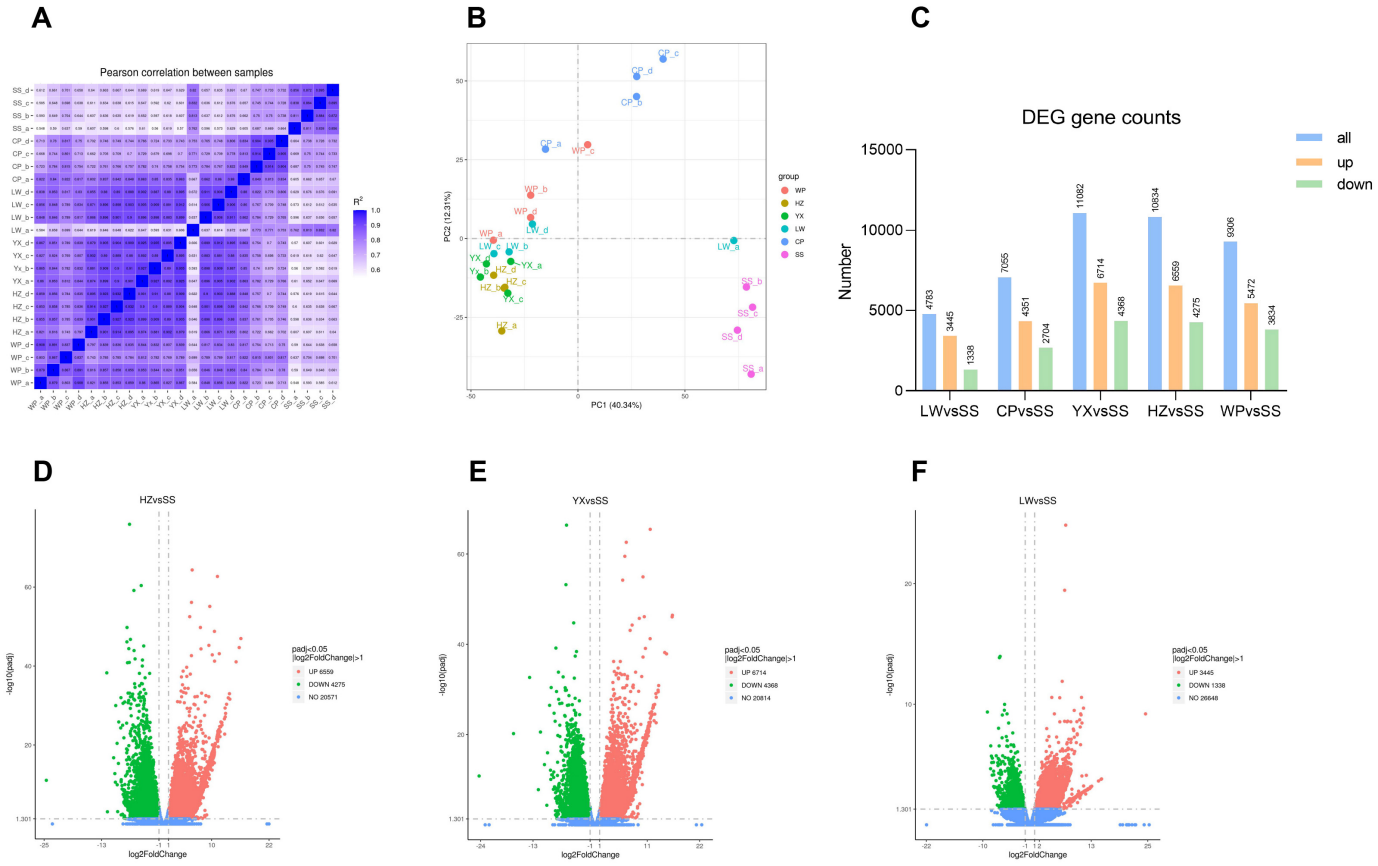
RNA-seq data analysis of mosquitoes collected from different geographical regions. **(A)** Correlation matrix of samples. **(B)** Principal component analysis (PCA) plot. **(C)** Screening of differentially expressed genes (DEGs) for each group compared to the SS group. **(D–F)** Volcano plots of DEGs for the comparisons HZ vs. SS, YX vs. SS, and LW vs. SS.

Quality assessment of the sequencing data showed that all samples met the requirements for subsequent analyses (Supplementary Table 1). To evaluate experimental reproducibility and transcriptome differences among samples, this study performed inter-sample correlation analysis and principal component analysis (PCA). The Pearson correlation heatmap of inter-sample relationships (Figure 1A) showed that the correlation coefficients of intra-group samples (all > 0.95) were significantly higher than those of inter-group samples, indicating good experimental reproducibility and reasonable sample grouping. The results of principal component analysis (PCA) (Figure 1B) further verified the above conclusion: the four biological replicates within each experimental group clustered closely together, reflecting good intra-group reproducibility; the laboratory susceptible strain (SS) was clearly separated from other field populations along the PC1 axis, suggesting global gene expression differences among populations.

### 3.4 Differential gene analysis and enrichment analysis

**Figure 2:**
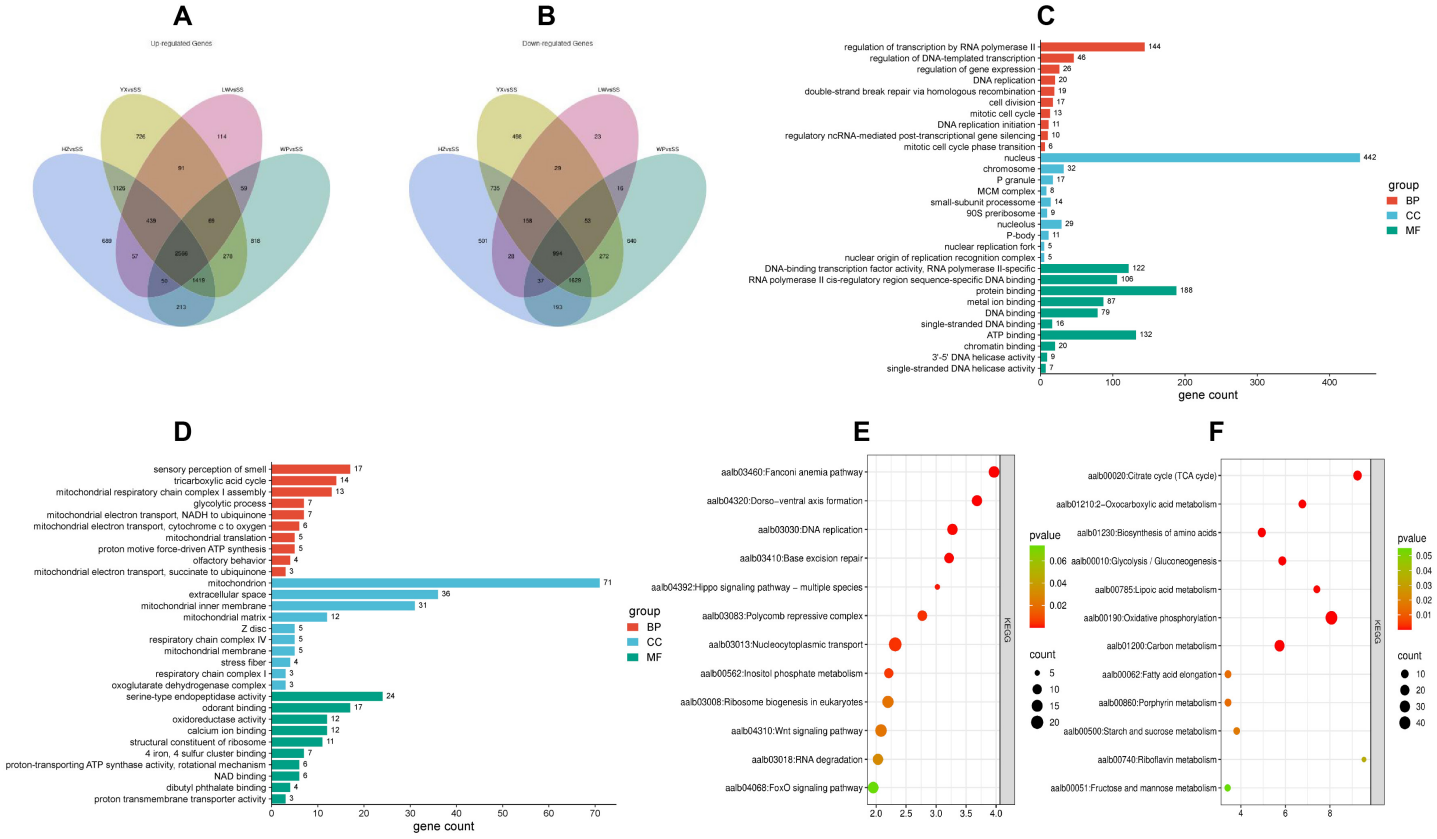
Venn diagrams and enrichment analysis of differentially expressed genes (DEGs). **(A, B)** Venn diagrams showing the numbers of up- and down-regulated DEGs shared among the HZ vs SS, YX vs. SS, LW vs. SS, and WP vs. SS comparisons. **(C, D)** Gene Ontology (GO) functional enrichment analysis of DEGs from the four comparison groups. **(E, F)** Kyoto Encyclopedia of Genes and Genomes (KEGG) pathway enrichment analysis of DEGs from the four comparison groups.

Differential expression analysis was performed using DESeq2 (v1.20.0) software with the criteria of FDR < 0.05 and |log_2_FC| > 1, and a large number of differentially expressed genes (DEGs) were detected in each comparison group. Compared with the susceptible strain (SS), 6559/4275, 6714/4365, 3445/1336, 5472/3834 and 4351/2704 up-/down-regulated genes were identified in HZ, YX, LW, WP and CP populations, respectively (Figures 1C–F). To focus on the core transcriptional signatures associated with β-cypermethrin resistance, Venn diagram analysis was further conducted on the common DEGs between the four resistant populations (HZ, YX, LW, WP) and SS, which identified a total of 2,566 common up-regulated genes (Figure 2A) and 994 common down-regulated genes (Figure 2B).

GO enrichment analysis showed that the common up-regulated genes were mainly involved in biological processes such as transcriptional regulation (e.g., RNA polymerase II-mediated transcription), DNA replication and repair, and cell cycle. The encoded proteins were mostly localized to the nucleus, chromosomes, and macromolecular complexes (e.g., MCM complex), and possessed molecular functions including transcription factor binding, DNA/RNA binding, and ATP binding (Figure 2C). In contrast, the common down-regulated genes were significantly enriched in processes such as olfactory perception, tricarboxylic acid cycle, and mitochondrial respiratory chain. Their products were mostly distributed in the inner mitochondrial membrane and matrix, with molecular functions involving serine-type endopeptidase activity, odorant binding, and oxidoreductase activity (Figure 2D).

KEGG pathway analysis further revealed that the most significantly enriched pathway among the common up-regulated genes was the Fanconi anemia pathway (aalb03460) (Figure 2E), whereas the common down-regulated genes were mainly enriched in the citrate cycle (TCA cycle, aalb00020) (Figure 2F), suggesting a potential association between attenuated mitochondrial energy metabolism and the resistant populations.

To clarify the transcriptional levels of target-site resistance genes in different populations, the expression data of the *kdr* gene (voltage-gated sodium channel, VGSC; gene ID: LOC109421922) were extracted from the expression matrix normalized by the DESeq2 software. The transcript counts of the *kdr* gene in each population were calculated and averaged for comparative analysis with the susceptible strain SS (Table 6). The results showed that the transcript abundance of *kdr* was significantly lower in the WP population (log_2_ FC = −3.04, padj = 0.00045) and HZ population (log_2_ FC = −1.84, padj = 0.040) than in the susceptible strain SS. In contrast, no significant differences in *kdr* transcript abundance were observed in the YX population (log_2_ FC = −1.67, padj = 0.085), LW population (log_2_ FC = −1.24, padj = 0.287), and CP population (log_2_ FC = −1.59, padj = 0.092). These results indicated that transcriptional variation of the kdr gene was unlikely to be the main factor associated with β-cypermethrin resistance in most populations. In contrast, the strong correlation between kdr mutation frequency and resistance phenotype suggests that target-site mutations play a more significant role in the observed resistance phenotypes.

**Table 6:** Expression level and differential significance of the *kdr* gene in different geographical populations of *Ae. Albopictus*.

Population	TPM/FPKM	log_2_ fold change vs. SS	*p*-value	padj
YX	27.89	−1.67	0.0423	0.0852
WP	10.32	−3.04	7.58e^−5^	0.000447
LW	33.32	−1.24	0.1410	0.2870
HZ	26.98	−1.84	0.0178	0.0403
CP	25.72	−1.59	0.0348	0.0922

log_2_FoldChange < 0 indicates lower *kdr* expression in field populations than in the SS strain.

### 3.5 Analysis of mosquito gut microbiota community richness and diversity

To elucidate the differences in gut microbiota among *Ae. albopictus* populations from different geographical locations, full-length 16S rRNA amplicon sequencing was performed on the mosquito midgut microbiota. After CCS correction, barcode splitting and primer trimming, a total of 475,294 high-quality sequences (mean length: 1,442.5 nt, Supplementary Table 2) were obtained for subsequent analyses.

Analysis of α-diversity revealed differences in the microbial community structure among populations (Table 7). For richness indices, the CP population had the highest Observed ASVs and the LW population the lowest; notably, the HZ population with high pyrethroid resistance exhibited significantly lower richness than the CP, WP and YX populations. The Shannon diversity index was the highest in the WP population, followed by the CP population. These results indicated that pyrethroid resistance in *Ae. albopictus* may be associated with the abundance of specific microbial taxa, rather than the overall community size or diversity. Therefore, subsequent analyses will focus on β-diversity analysis and identification of key microbial genera to further screen for microbial taxa associated with the resistant phenotype.

**Table 7:** Alpha diversity analysis of mosquito gut bacterial communities in different regions.

Group	Community richness		Community diversity	
	Chao1	Ace	Shannon	Simpson
HZ	110.38 ± 85.18	120.55 ± 70.71	1.4 ± 0.14	0.43 ± 0.06
YX	463.66 ± 97.44	490.52 ± 80.13	3.53 ± 0.85	0.73 ± 0.1
LW	54.17 ± 20.33	49.24 ± 17.01	0.27 ± 0.07	0.06 ± 0.02
CP	722.65 ± 186.93	718.42 ± 162.72	5.08 ± 0.84	0.85 ± 0.07
WP	635.43 ± 77.13	602.35 ± 58.97	5.48 ± 0.08	0.86 ± 0.01
SS	603.14 ± 135.37	590.05 ± 135.95	4.96 ± 0.67	0.88 ± 0.05

### 3.6 Microbial community composition of the mosquito gut

**Figure 3:**
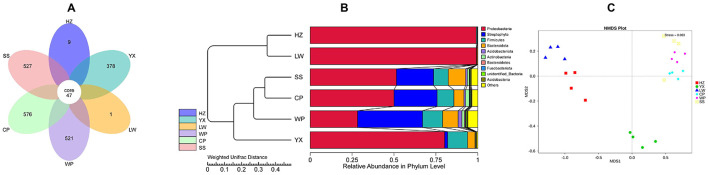
Composition of the intestinal bacterial community in mosquitoes from different regions. **(A)** Flower plot (Venn diagram) showing the overlap of operational taxonomic units (OTUs) among different groups. **(B)** WPGMA clustering tree based on the evolutionary relationships of intestinal bacterial communities, with the bar plot showing the relative abundance of dominant phyla. **(C)** Non-metric multidimensional scaling (NMDS) plot illustrating the beta diversity among samples from different groups.

To elucidate the differences in the composition and structure of gut microbiota among different *Ae. albopictus* populations, we conducted OTU and diversity analyses. The OTU clustering results showed that there were a total of 47 shared OTUs across the six sample groups, among which the CP, WP and SS groups had a large number of unique OTUs (576, 521 and 527, respectively), while the HZ group had only 9 unique OTUs (Figure 3A).

The WPGMA clustering tree further divided the samples into two subgroups (Figure 3B): the highly resistant HZ and LW populations clustered in the same clade, the susceptible SS and CP populations clustered in another clade, and the YX population was distributed independently. At the phylum level, significant differences were observed in the microbial community composition among the populations. The communities of the HZ and LW populations were highly specialized, with the relative abundance of Proteobacteria exceeding 99%. The LW population exhibited extremely low diversity, and multiple phyla were not detected. In contrast, the microbial community composition of the SS, CP and WP populations was more abundant and balanced, consistent with the results of α-diversity analysis.

NMDS analysis showed (Figure 3C) that intra-group samples clustered closely with distinct inter-group separation. The HZ, YX and LW populations were far from the susceptible control SS, while the CP and WP populations clustered with SS, indicating high similarity in their microbial community structures.

**Figure 4:**
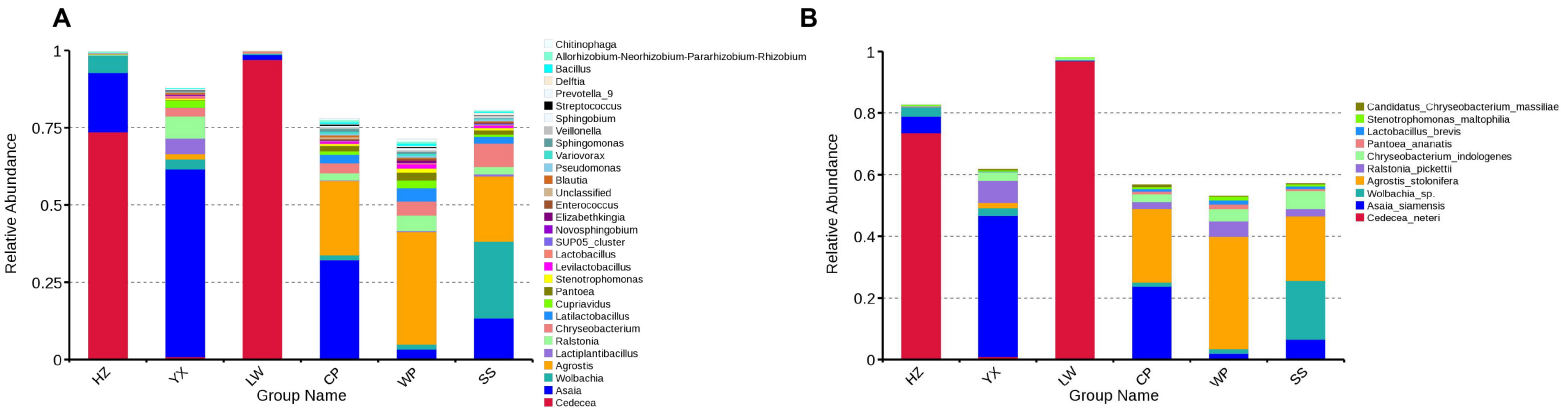
Relative abundance of dominant bacterial taxa in the midgut of *Ae. albopictus*. **(A)** Top 30 bacterial genera. **(B)** Top 10 bacterial species.

To identify microbial taxa associated with pyrethroid resistance, we further analyzed the midgut microbiota composition at the genus and species levels. Genus-level analysis (Figure 4A) showed that *Cedecea* was dominant in the HZ population with the highest resistance, suggesting a potential positive association between this genus and high resistance level and also exhibited high abundance in the YX population; these microbes are considered candidate taxa potentially associated with pyrethroid resistance. *Asaia* abundance was significantly elevated in resistant populations such as HZ and YX; whereas Wolbachia was mainly distributed in susceptible populations and moderate-to-low resistant populations including WP, with extremely low abundance in highly resistant populations, suggesting a potential negative correlation with the resistant phenotype.

Species-level results further confirmed the above trends (Figure 4B). *Cedecea neteri* was the key species driving the high abundance of this genus in the HZ and LW populations; *Asaia siamensis* was specifically elevated in the YX and CP populations; and *Wolbachia sp*. showed the highest abundance in susceptible and WP populations, and was almost undetectable in highly resistant populations.

In summary, pyrethroid resistance in *Ae. albopictus* may be associated with the relative abundance of specific midgut microbes (e.g., *Cedecea neteri, Asaia siamensis* and *Wolbachia sp*.), these microbes are identified as key candidate taxa for pyrethroid resistance. These taxa provide key candidate targets for subsequent validation of microbe-mediated resistance mechanisms.

### 3.7 Correlation analysis between mosquito gene expression and gut microbiota abundance

To elucidate the differences in the composition and structure of gut microbiota among different *Ae. albopictus* populations, we conducted OTU and diversity analyses. The OTU clustering results showed that there were a total of 47 shared OTUs across the six sample groups, among which the CP, WP and SS groups had a large number of unique OTUs (576, 521 and 527, respectively), while the HZ group had only 9 unique OTUs (Figure 3A). The WPGMA clustering tree further divided the samples into two subgroups (Figure 3B): the highly resistant HZ and LW populations clustered in the same clade, the susceptible SS and CP populations clustered in another clade, and the YX population was distributed independently.

**Figure 5:**
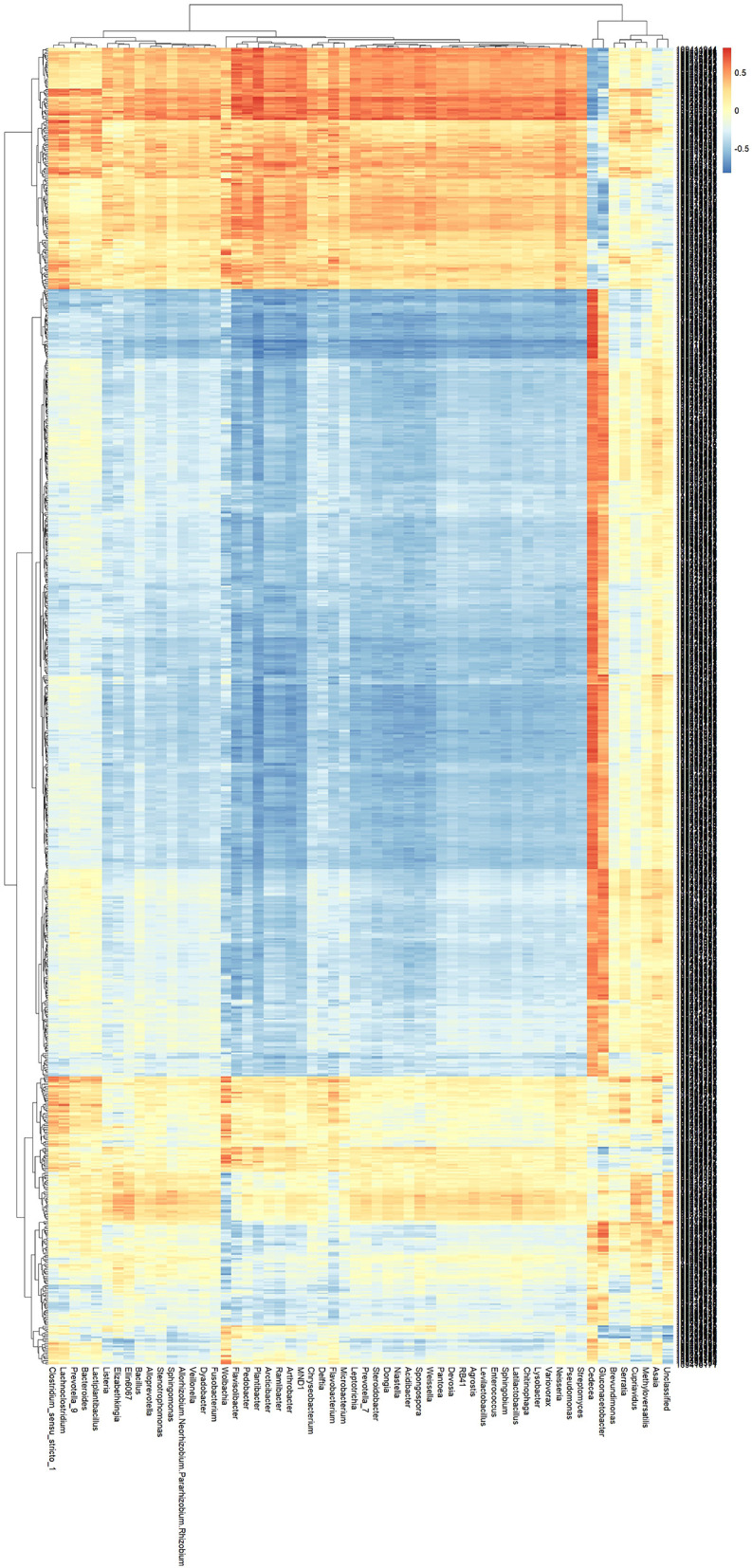
Correlation heatmap between insecticide resistance-related differentially expressed genes (DEGs) and differential microbial genera in *Ae. albopictus*. The heatmap displays the Spearman correlation coefficients between 838 resistance-related DEGs (columns) and 68 microbial genera with differential abundance (rows). The color scale ranges from blue (strong negative correlation) to red (strong positive correlation), with yellow indicating no correlation. The highlighted genera *Wolbachia, Gluconacetobacter, Cedecea, Flavisolibacter, Plantibacter*, and *Asaia* exhibit the most extensive correlations with DEGs, as visualized by dense clusters of colored cells.

Based on 16S rRNA gene sequencing data, 68 microbial genera with significantly differential abundance were identified between field pyrethroid-resistant populations and susceptible strains of *Ae. albopictus*. Using Spearman correlation analysis, we further constructed a co-occurrence association network between 838 drug resistance-related differentially expressed genes (DEGs) and these 68 microbial genera, with the results of the association analysis shown in Supplementary Table 4. It can be clearly seen from the heatmap clustering analysis (Figure 5) and the association analysis in Supplementary Table 4 that six core genera exhibited the closest regulatory associations with drug resistance-related DEGs. Specifically, the six core genera, namely *Wolbachia, Gluconacetobacter, Cedecea, Flavisolibacter, Plantibacter*, and *Asaia*, showed significantly stronger association strength with drug resistance-related DEGs than other genera, and their correlation levels presented distinct red/blue clustering regions in the heatmap. According to Supplementary Table 4, each of these core genera was significantly correlated with 28–45 DEGs at an absolute correlation coefficient greater than 0.6, which was remarkably higher than other microbial genera (correlated with only 5–8 DEGs on average). Furthermore, the association analysis revealed that these core genera were preferentially associated with key drug resistance pathway genes. For instance, *Cedecea* was positively correlated with the cytochrome P450 metabolic gene CYP6P5 with a correlation coefficient of 0.82, and with the glutathione S-transferase gene GSTe2 with a coefficient of 0.76; Wolbachia was correlated with the ABC transporter gene ABCG4 with a coefficient of 0.79. The associations of these key genes appeared as dense clustering regions in the heatmap, further verifying the tight regulatory relationships between these genera and the expression of drug resistance-related genes.

In conclusion, the six core genera (*Wolbachia, Gluconacetobacter, Cedecea, Flavisolibacter, Plantibacter*, and *Asaia*) may be associated with the development of the pyrethroid-resistant phenotype in *Ae. albopictus* by regulating the expression of genes related to metabolic detoxification, xenobiotic efflux, and target resistance.

Among these genera, the associations of *Wolbachia* and *Asaia* with mosquito resistance have been supported by published literature, whereas studies on *Gluconacetobacter* and *Cedecea* remain scarce. Notably, *Cedecea* was the dominant genus in the resistant HZ population with significantly higher abundance than in the susceptible strain. Therefore, subsequent experiments focused on the midgut enrichment of *Cedecea* to verify its impact on pyrethroid resistance.

**Figure 6:**
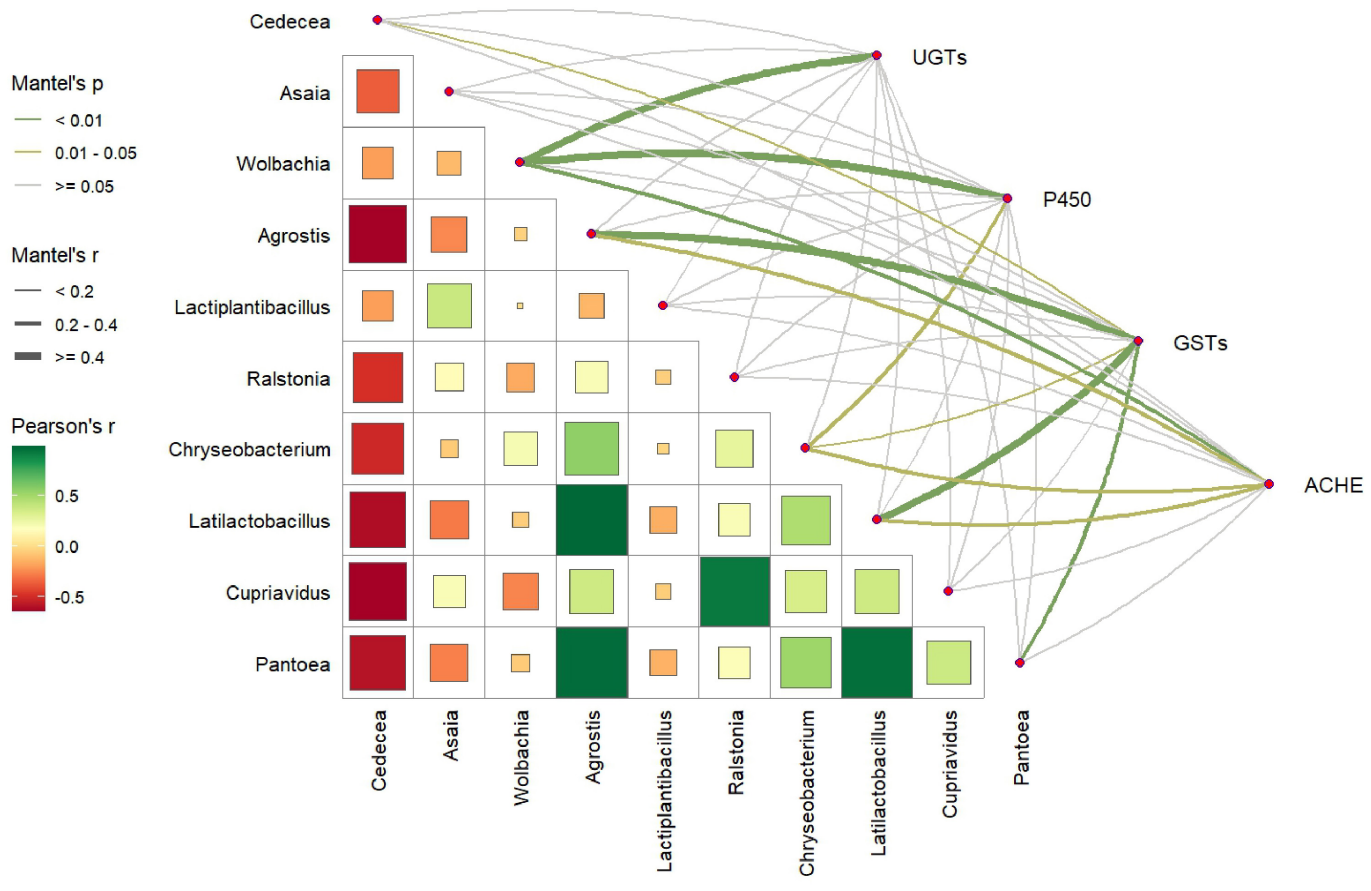
Correlation and Mantel test analysis.

To investigate whether the gut microbiota modulates mosquito resistance by regulating metabolic detoxification pathways, we focused on genes related to the metabolism of xenobiotics and pharmaceuticals, and identified a total of 414 genes (Supplementary Table 5), including 34 UGT, 297 P450, 59 GST and 24 AChE genes. Further analysis of the correlations between these genes and the top 10 microbial genera with the highest abundances in the gut microbiota (Figure 6) revealed a negative correlation among most genera, with only a few combinations (e.g., *Latilactobacillus* and *Chryseobacterium, Cupriavidus* and *Pantoea*) showing strong positive correlations, implying potential synergistic and antagonistic relationships of the midgut microbiota in stress responses.

Gene-microbiota correlation analysis indicated that different detoxification gene families were significantly correlated with specific microbial genera. UGT genes were correlated with *Wolbachia*; P450 genes were correlated with *Wolbachia* and *Chryseobacterium*; GST genes were correlated with *Cedecea, Agrostis, Chryseobacterium, Latilactobacillus* and *Pantoea*; and AChE genes were correlated with *Wolbachia, Agrostis, Chryseobacterium* and *Latilactobacillus*. These results suggest that the gut microbiota may affect mosquito resistance by specifically regulating the expression of host detoxification genes.

### 3.8 Verification of the correlation between mosquito gut microbiota and pyrethroid resistance

This study is based on the screening of dominant bacterial strains from insect populations resistant to pyrethroids and multi-omics association analysis, and identified *Cedecea* as a candidate bacterial genus for subsequent supplementation and resistance validation. *Cedecea neteri* bacterial suspension (10^9^ CFU) was orally administered for 24 h to construct a *Cedecea neteri* supplemented group of *Ae. albopictus*, with a control group fed with sterile sugar solution.

**Figure 7:**
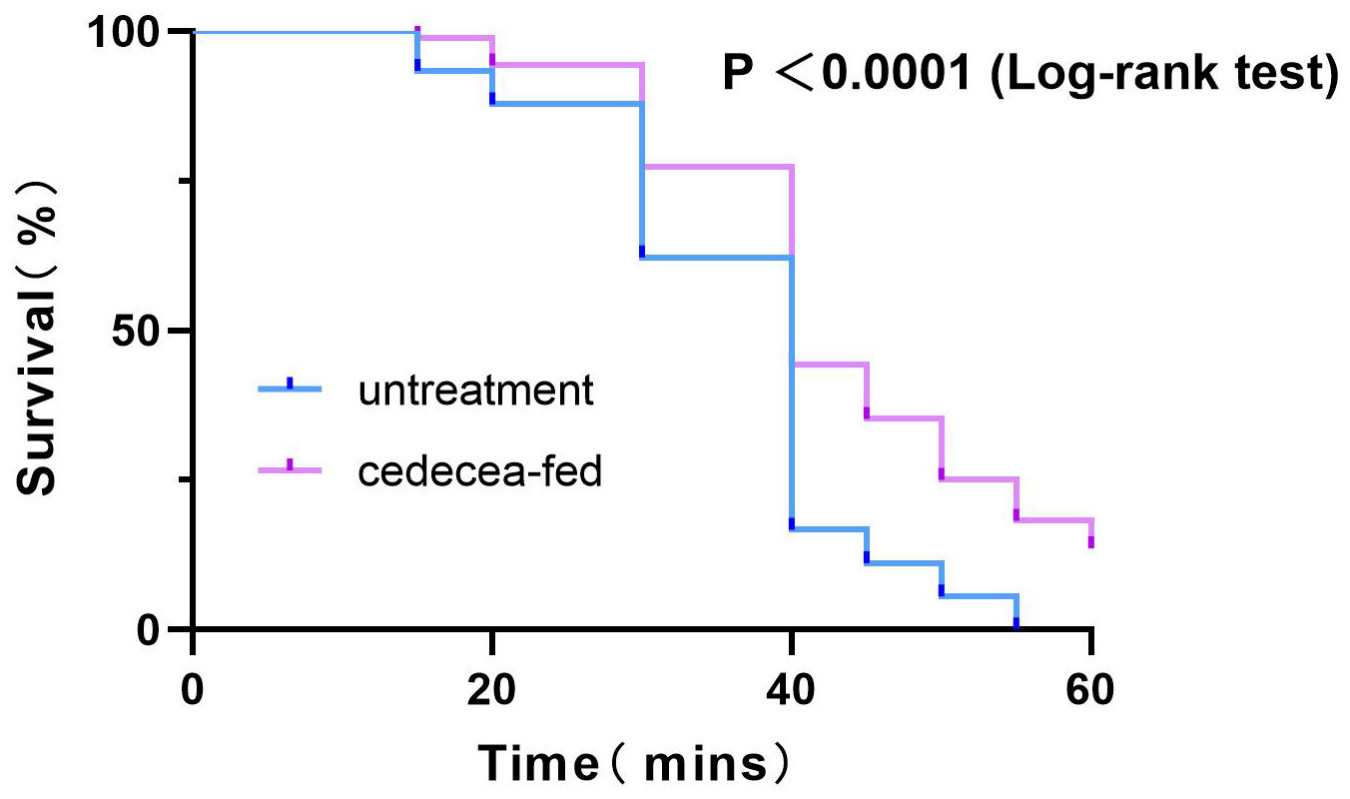
Survival curves of *Ae. albopictus* exposed to 0.1 mg/mL β-cypermethrin within 1 h. Three independent biological replicates were conducted, with 30 mosquitoes in each replicate. The difference between groups was analyzed by the log-rank test, which showed a statistically highly significant difference (*P* < 0.0001).

A CDC bottle bioassay was conducted with a contact pesticide concentration of 0.1 mg/L deltamethrin. Two sets of data were collected to evaluate the effect of *Cedecea neteri* supplementation on pyrethroid resistance: (1) Time-dependent survival dynamics during pesticide exposure: Kaplan–Meier survival curves were plotted to monitor the survival of mosquitoes at multiple time points during a 1-h exposure to 0.1 mg/L deltamethrin (Figure 7). During the exposure period, mosquitoes that were knocked down and unable to stand or fly normally were considered temporarily dead. The results showed that, compared to the untreated control group, the Cedecea net*eri* supplementation group had significantly higher survival rates (Log-rank test, *P* < 0.0001). Each group contained three biological replicates (30 individuals per replicate, total sample size *n* = 90). (2) 24-h post-exposure survival rate: After a 60-min exposure in deltamethrin-treated bottles, mosquitoes were transferred to recovery cups, and the survival status was recorded after 24 h. The survival status was recorded after 24 h, where only mosquitoes that recovered normal standing and flying ability were considered surviving (some mosquitoes not truly killed by deltamethrin could recover during the 24-h recovery period). The results indicated that the survival rate of the *Cedecea neteri* supplementation group was 73.86%, significantly higher than the control group's survival rate of 40.00% (Fisher's exact test, *P* < 0.0001).

The above results suggest a significant association between the exogenous *Cedecea neteri* supplementation and the enhanced pyrethroid resistance in *Ae. albopictus*.

## 4 Discussion

The development of resistance to pyrethroid insecticides in *Ae. albopictus* poses a major challenge to the current prevention and control of mosquito-borne infectious diseases (Näslund et al., 2021; Ahmad et al., 2020). In the absence of specific drugs and vaccines for diseases such as dengue fever, chemical control remains the primary intervention, yet the evolution of resistance has severely compromised its efficacy (Abbasi, 2025). In this study, field populations of *Ae. albopictus* collected from Guangdong and Hainan Provinces of China were assayed. Results showed that all populations from Guangdong exhibited a certain level of resistance to β-cypermethrin, with the Haizhu Park population displaying the highest resistance (LC_50_ was 29.18-fold that of the laboratory susceptible strain), which is closely associated with the extensive use of pyrethroid insecticides in this region (Su et al., 2019). In contrast, populations from Hainan showed low resistance or remained susceptible, possibly due to the lower frequency of insecticide application and slower resistance development locally. This finding is consistent with previous studies and also indicates that resistance evolution is directly related to insecticide selection pressure (Zhao et al., 2023; Li et al., 2020, 2021).

Target-site mutations in the voltage-gated sodium channel (VGSC) gene, known as *kdr* mutations, are a classic mechanism of resistance to pyrethroid insecticides in mosquitoes. In this study, three mutations (F1534C, F1534L, F1534S) at codon 1,534 of the VGSC gene were detected in all field populations, with mutation frequencies ranging from 49.4% [CP population (CP: Haikou Shishan Volcanic Cluster Global Geopark)] to 100.0% [HZ population (HZ: Haizhu Lake Area (Guangzhou)]. In contrast, only one mutation (V1016G) was detected at codon 1,016, with a low frequency (0%~20.7%), and this mutation was not even detected in the WP population and SS strain. Notably, the HZ population, which had the highest resistance level, also had the highest *kdr* mutation frequency (100.0% for codon 1,534), while the relatively susceptible CP population had the lowest mutation frequency (49.4%). This strong positive correlation between *kdr* mutation frequency and resistance level indicates that codon 1,534 substitutions are strongly associated with β-cypermethrin resistance in the tested populations, which is consistent with previous reports that F1534 mutations are the most prevalent and impactful *kdr* mutations in *Ae. albopictus* (Chatterjee et al., 2018; Aguirre-Obando et al., 2022). In addition, we found no significant differences in the transcriptional level of the VGSC gene in most populations, indicating that target-site mutation is the main genetic signature associated with target-site resistance in these field populations, rather than transcriptional regulation of the VGSC gene.

Transcriptome analysis revealed a total of 2,566 up-regulated and 994 down-regulated genes in the resistant populations. The up-regulated genes were mainly enriched in processes including transcriptional regulation, DNA repair and cell cycle, whereas the down-regulated genes were associated with olfactory perception, tricarboxylic acid cycle and mitochondrial function, reflecting metabolic reprogramming during resistance development. Notably, multiple up-regulated genes closely associated with β-cypermethrin resistance were identified, including Cytochrome P450 monooxygenase genes (CYP6A1, CYP6A14, CYP6D5, CYP9E2, CYP18A1) and glutathione S-transferase genes (GST D1, GST 1D, GST 1B). These genes play crucial biological roles in the metabolic detoxification of insecticides: Cytochrome P450 monooxygenases are key enzymes involved in the oxidation and detoxification of pyrethroids, while glutathione S-transferases catalyze the conjugation of glutathione with toxic metabolites, thereby enhancing the degradation and excretion of β-cypermethrin in mosquitoes. Their up-regulation is considered to be potentially involved in strengthening the degradation ability of mosquitoes against β-cypermethrin, thereby contributing to the development of resistance. Pathway analysis further demonstrated that detoxification-related pathways such as glutathione metabolism, nucleotide metabolism and ABC transporters were significantly activated (Messenger et al., 2021; Bariami et al., 2012; Lv et al., 2016); meanwhile, the up-regulation of the Fanconi anemia pathway suggested that DNA damage repair plays an important role in resistance adaptation. Collectively, these results suggest that the resistance phenotype involves multi-level regulation of gene expression and metabolic reorganization.

This study further revealed that the gut microbial community structure of pyrethroid-resistant *Ae. albopictus* populations was significantly altered, with their α-diversity generally lower than that of susceptible populations, and Proteobacteria dominating absolutely in the highly resistant populations. Through multi-omics correlation analysis, we constructed a gene-microbe interaction network and found that microbial genera including *Cedecea, Asaia* and *Wolbachia* were significantly correlated with a large number of resistance-related genes. Among these, the association of *Wolbachia* with UGT, P450 and AChE genes suggested that it may modulate mosquito resistance by regulating the expression of host detoxification and target enzymes. In contrast, the strong correlation between *Cedecea* and GST genes indicated that this genus may be involved in resistance development by regulating the antioxidant stress pathway. Notably, through mosquito feeding experiments, we found that *Cedecea neteri* could significantly improve the survival rate of *Ae. albopictus* under the stress of β-cypermethrin, suggesting that this bacterial strain is associated with increased tolerance to β-cypermethrin of *Ae. albopictus* to this insecticide to a certain extent.

In addition, although *Asaia* showed no strong statistical correlation with core detoxification genes in this study, previous research has demonstrated that it can directly metabolize pyrethroids via its harbored pesticide-degrading enzyme genes (Comandatore et al., 2021; Djondji Kamga et al., 2023), suggesting that microbe-mediated resistance exhibits functional diversity, and some microbial genera may exert independent effects without relying on host gene regulation.

Some limitations of this study deserve attention. First, transcriptomic analysis was performed on mosquito samples prior to RNA extraction without prior exposure to insecticides. Therefore, the observed differential gene expression patterns represent baseline transcriptional differences rather than inducible responses to insecticide stress. This means that these patterns may not only reflect resistance-associated traits but also include geographical variation, environmental adaptation, or inherent differences between field and laboratory-reared populations. Consequently, we subsequently focused primarily on detoxification metabolism-related genes for network construction and analysis. Second, this study only conducted functional validation of Cedecea neteri and did not verify its colonization in the mosquito midgut. The roles of other key bacterial genera (e.g., *Asaia, Wolbachia*) in insecticide resistance still require further investigation. Third, the specific molecular mechanisms by which *Cedecea neteri* regulates host detoxification genes or directly degrades β-cypermethrin remain unclear and need to be further explored in future studies. Fourth, laboratory rearing conditions may alter the original microbial characteristics of field populations. Although microbial differences among populations were still detected, they cannot fully reflect the authentic structure of microbial communities in the wild.

In summary, this study demonstrates that resistance to β-cypermethrin in *Ae. albopictus* is a complex phenotype driven jointly by host genetic factors (*kdr* mutations and gene expression reprogramming) and the remodeling of gut microbial communities. Although standardized laboratory rearing reduced environmental noise and allowed clearer observation of microbial differences likely driven by host genetic background, it may also have diminished the unique microbiota shaped by natural field environments. The observed microbial differences among F1 populations in this study likely result from the combined effects of vertically transmitted heritable microbes and selective pressure exerted by host genotype on the microbiota, providing a new perspective for understanding biological differences between field and laboratory populations. By integrating transcriptomic and microbiomic analyses, we systematically identified key resistance-associated genes and microbial taxa, and preliminarily confirmed that supplementation with *Cedecea neteri* is associated with enhanced resistance in *Ae. albopictus*. These findings provide a theoretical basis for further clarifying the microbe–host interaction mechanisms underlying insecticide resistance and for developing novel green control strategies against *Ae. albopictus* (e.g., targeting gut microbiota to reverse or slow resistance evolution). Future research should focus on functional verification of other key microbial strains, validation of Cedecea neteri colonization in the mosquito midgut, specific molecular pathways of microbe–host interactions, and the ecological stability of microbe-mediated resistance in natural field populations.

## Data Availability

The datasets presented in this study can be found in online repositories. The names of the repository/repositories and accession number(s) can be found in the article/Supplementary material.
