# Supplementary material for: Integrated transcriptome-microbiome analysis reveals a host-microbe interplay associated with insecticide resistance in Aedes albopictus
Source: Front Microbiol. 2026 Apr 22;17:1788609. doi: 10.3389/fmicb.2026.1788609 (PMC13143895; doi:10.3389/fmicb.2026.1788609)
Supplement: Supplementary file 6 [file Table_6.docx]

Supplementary Material

# Supplementary Data

The sequencing data have been submitted to the NCBI SRA under BioProject PRJNA1401450 and will be publicly available upon publication of this article.

# Supplementary Tables

**Supplementary Table 1.** Summary of sample sequencing data quality.

**Supplementary Table 2.** Data preprocessing statistics and quality control.

**Supplementary Table 3.** Correlation analysis between differential gene expression levels and abundance of differential bacterial genera.

**Supplementary Table 4.** Correlation analysis.

**Supplementary Table 5.** Expression levels of genes related to metabolic detoxification enzymes.
